# Genetic code robustness and protein evolvability are correlated and protein-specific

**DOI:** 10.1371/journal.pbio.3002627

**Published:** 2024-05-17

**Authors:** Brian P. H. Metzger

**Affiliations:** Department of Biological Sciences, Purdue University, West Lafayette, Indiana, United States of America

## Abstract

The relationship between genetic code robustness and protein evolvability is unknown. This Primer explores a new PLOS Biology study which uses in silico rewiring of genetic codes and functional protein data to identify a positive correlation between code robustness and protein evolvability that is protein-specific.

## Teaser

The relationship between genetic code robustness and protein evolvability is unknown. A new study in *PLOS Biology* using in silico rewiring of genetic codes and functional protein data identified a positive correlation between code robustness and protein evolvability that is protein-specific.

The standard genetic code is a central feature of life on our planet, providing a nearly universal mechanism to translate DNA sequence into corresponding amino acids. Since its decoding in the early 1960s, questions have been asked about the evolutionary origins of the standard genetic code and what properties contributed to its emergence [1,2]. In particular, there are more than a quintillion (20 factorial or approximately 10^18^) possible genetic codes with the same basic structure as the standard genetic code—the same number of amino acids, codon blocks, split codons (where an amino acid is encoded by disjoint codons), and the location of stop codons—but with different codon blocks coding for different amino acids. Given this immense set of alternative genetic codes, what, if anything, is special about the standard genetic code?

The structure of the genetic code influences evolution. For example, single-nucleotide mutations are the most common type of mutation during evolution, but the standard genetic code uses triplet codons, and thus some codons are multiple mutational steps away from one another. In addition, the existence of stop and synonymous codons, and the organization of synonymous codons by the third nucleotide position, means that each codon in the standard genetic code has on average fewer than 6 neighboring codons that differ by a single nucleotide yet code for a different amino acid. Since amino acids have different physiochemical properties (such as size, charge, and hydrophobicity), and certain amino acids exhibit greater similarity than others, which amino acids can directly mutate into one another determines which physiochemical changes are most likely to arise via mutation during evolution. It has been proposed that the standard genetic code is more robust than other genetic codes because mutations under the standard genetic code are less likely to alter the physiochemical properties of amino acids than under other genetic codes [3]. Furthermore, this robustness has been proposed to be a key feature promoting the origin of the standard genetic code over alternatives because it would be evolutionarily beneficial for a genetic code to increase the likelihood that a mutation is tolerated by natural selection [4].

**Fig 1:**
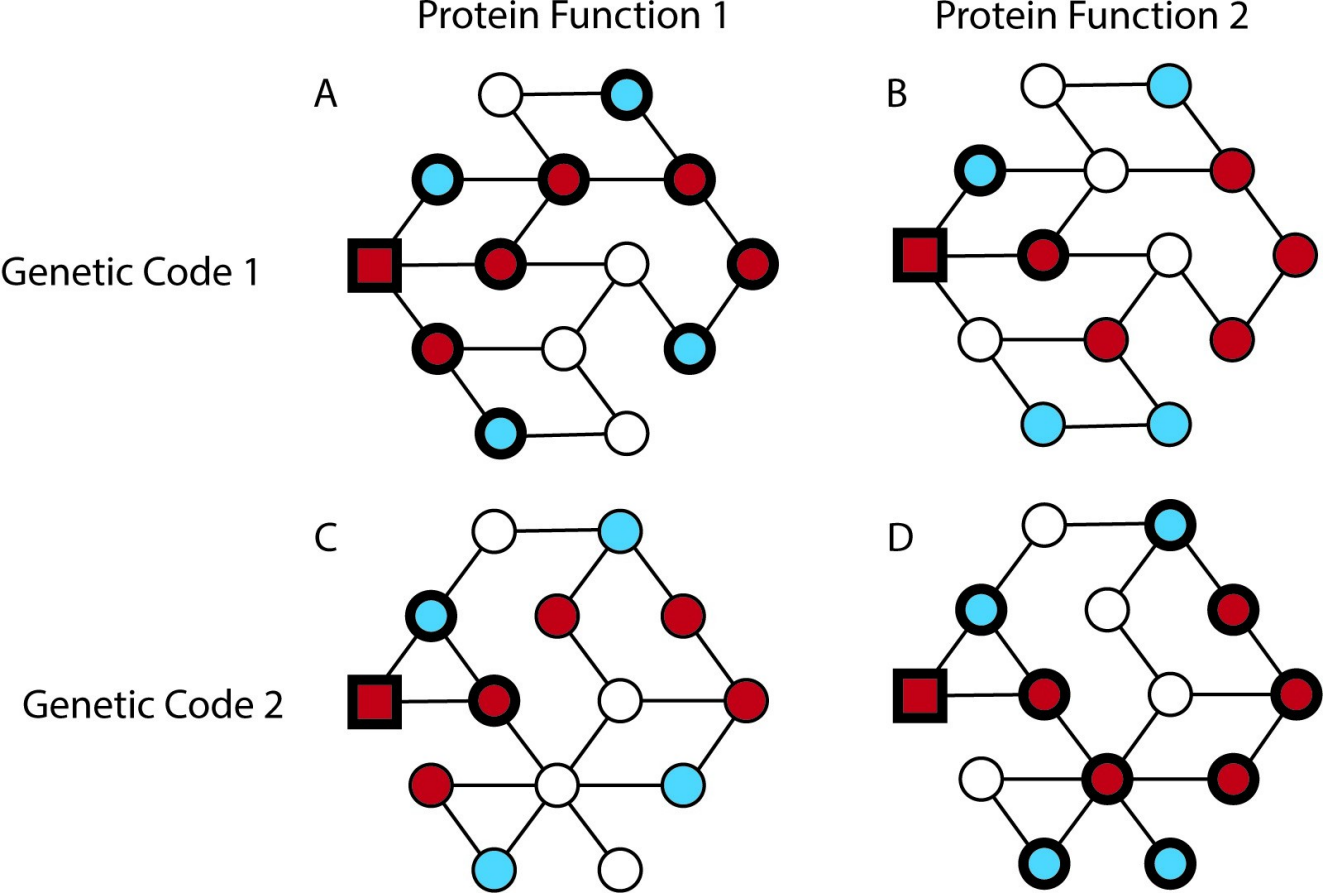
Robust genetic codes confer more protein evolvability, but these properties can be protein-specific. Example networks relating robustness, evolvability, the genetic code, and different protein functions. Each circle represents a unique sequence, with color denoting its function: red for functional, white for nonfunctional, and blue for increased function. Sequences are connected if a single-nucleotide mutation can transform one into the other according to the specific genetic code being used. Natural selection will typically prevent significant decreases in function. Thus, the ability to find adaptive variation (evolvability, bold blue circles) from a particular staring point (red square) is influenced by the size of the network of sequences with similar function (robustness, bold red circles). Diagrams in the same column have the same distribution of protein functions on the network. Diagrams in the same row use the same genetic code, and thus connections among neighboring sequences. (**A)** A robust and evolvable function under a particular genetic code. (**B)** The same genetic code may provide less robustness and less evolvability for a different protein function. (**C)** A protein function that is robust under one genetic code may not retain this property under an alternative code. (**D)** This alternative genetic code may increase robustness and evolvability for a different protein function. Thus, a positive correlation between genetic code robustness and evolvability can also lead to differences among which genetic codes are the most robust or confer the most protein evolvability for a particular protein function.

While a robust genetic code may be advantageous, evolution’s ability to generate new functions and adaptive variation, known as evolvability, is also important. Robustness and evolvability are often perceived as inversely related, as robustness to mutations implies there are few mutations that cause functional changes, seemingly at odds with evolvability [5]. However, work on a variety of systems, such as RNA secondary structures [6] and protein–DNA interactions [7], has shown that robustness can facilitate evolvability under certain conditions [8] (Fig 1). Specifically, robustness to mutations can lead to numerous sequences with similar functions that differ by few mutations. This creates a network of sequences that can be readily explored by evolution since many mutations have little impact on function. At the same time, because the sequences in the network are not identical, they harbor distinct neighbors, some of which may possess altered functions or yield adaptive mutations. Thus, the broader the network of sequences with similar functions, i.e., the more robust sequence function is to mutation, the greater the likelihood that some sequence neighboring the network will contain a novel function or adaptive variant. The relationship between robustness and protein evolvability within the standard genetic code and whether this relationship is more pronounced than expected under the myriad of alternative genetic codes remain open questions.

The new work by Rozhoňová and colleagues set out to empirically address these questions [9]. The authors first introduced a metric of amino acid similarity that combines multiple physiochemical properties, departing from prior work that focused on individual properties. They then calculated the robustness of the standard genetic code as the proportion of single-nucleotide mutations that did not alter this integrated metric. To determine the robustness of alternative genetic codes, they created 100,000 in silico permutations of which codons encode which amino acids, effectively rewiring the genetic code and altering which amino acids can readily mutate to one another. As anticipated from prior studies, the standard genetic code exhibited greater robustness than many alternative genetic codes. However, the standard genetic code was not exceptionally robust and thousands of more robust alternative genetic codes were readily found.

To determine the relationship between genetic code robustness and protein evolvability and how this relationship differs among different genetic codes, the authors used 6 recently collected deep mutational scanning datasets. Each dataset contains nearly all possible amino acid combinations at 3 to 4 sites (8,000 to 160,000 unique amino acid sequences) and the protein function of each sequence. To calculate evolvability, they created a network of DNA sequences, such that neighboring sequences differed by only a single nucleotide and then used both the standard genetic code and the 100,000 rewired genetic codes to determine the amino acid sequence of each DNA sequence. The authors then overlayed each network with the protein function data and used both network properties and evolutionary simulations to estimate protein evolvability under each genetic code. On average, they found that more robust genetic codes conferred greater protein evolvability than less robust genetic codes. However, protein evolvability under the standard genetic code varied considerably among the different datasets and protein functions. In addition, the relationship between genetic code robustness and protein evolvability was often weak across datasets. This led the authors to explore other factors that had large effects on protein evolvability, such as the size, or number of DNA sequences, that code for proteins with high fitness. In addition, they tested the sensitivity of their findings to the metric used to estimate robustness by examining over 500 individual amino acid physicochemical properties instead of their combined metric. They also directly estimated robustness from the deep mutational scanning data by determining which amino acids are exchangeable and which are not. This metric has the advantage of being dataset- and amino acid-specific, thereby avoiding reliance on general similarities among amino acids that may not apply in all contexts. Such analyses, along with alternative ways to rewire the genetic code that are experimentally accessible, all support a positive relationship between genetic code robustness and protein evolvability. This suggests that the observed relationship is not a result of the specific metrics used but instead represents a general relationship.

In summary, the study by Rozhoňová and colleagues establishes a positive relationship between genetic code robustness and protein evolvability. This relationship is typically weak, and genetic codes with similar levels of robustness can vary considerably in the degree of protein evolvability conferred. The limited experimental data currently available, however, means that there are several outstanding questions that are currently unanswerable. For example, we do not know whether the relationship between genetic code robustness and protein evolvability varies across a protein, and what, if any, site characteristics this might correlate with (e.g., protein secondary structure, buried versus surface exposed residues, or evolutionary rate). In addition, there is currently little experimental evidence relating code robustness and protein evolvability, but the recent experimental availability of alternative genetic codes means that directly testing this idea using laboratory or directed evolution experiments should soon be possible [10]. Similarly, which physicochemical properties of amino acids are typically the most important for function, and thus what, if any, properties the standard genetic code might have been selected for, could be addressed using deep mutational scanning experiments with alternative genetic codes that use noncanonical amino acids. Regardless of these answers, the current work does establish that which genetic codes are the most robust, which promote protein evolvability the most, and the relative ranking of the standard genetic code compared to these alternative codes varies among protein functions. Thus, while the standard genetic code appears to be both robust and confer protein evolvability in many cases, it is not universally remarkable for either property. Such a result may be due to natural selection optimizing over multiple physiochemical properties and differences in amino acid exchangeabilities among sites and proteins, or it may reflect the chance fixation of one of a myriad of possible genetic codes that was simply good enough.
